# Human Isogenic Cell Line Models for Neutrophils and Myeloid-Derived Suppressor Cells

**DOI:** 10.3390/ijms21207709

**Published:** 2020-10-18

**Authors:** Yuting Zhang, Emily Wilt, Xin Lu

**Affiliations:** 1 Integrated Biomedical Sciences Graduate Program, University of Notre Dame, Notre Dame, IN 46556, USA; yzhang49@nd.edu; 2 Department of Biological Sciences, Boler–Parseghian Center for Rare and Neglected Diseases, Harper Cancer Research Institute, University of Notre Dame, Notre Dame, IN 46556, USA; ewilt@nd.edu; 3 Tumor Microenvironment and Metastasis Program, Indiana University Melvin and Bren Simon Cancer Center, Indianapolis, IN 46556, USA

**Keywords:** neutrophil, myeloid-derived suppressor cell, PMN-MDSC, HL60, Jurkat, GM-CSF, IL6, all-trans retinoic acid, STAT3 inhibitor

## Abstract

Neutrophils with immunosuppressive activity are polymorphonuclear myeloid-derived suppressor cells (MDSCs) and may contribute to the resistance to cancer immunotherapy. A major gap for understanding and targeting these cells is the paucity of cell line models with cardinal features of human immunosuppressive neutrophils and their normal counterparts, especially in an isogenic manner. To address this issue, we employ the human promyelocytic cell line HL60 and use DMSO and cytokines (granulocyte macrophage-colony stimulating factor (GM-CSF) and interleukin 6 (IL6)) to induce the formation of either neutrophils or MDSCs. The induced MDSCs are CD11b^+^ CD33^+^ HLA-DR^−/low^ and are heterogeneous for CD15 and CD14 expression. The induced MDSCs abrogate IL2 production and activation-induced cell death of the human T cell line Jurkat stimulated by CD3/CD28 antibodies, whereas the induced neutrophils enhance IL2 production from Jurkat cells. The induced MDSCs upregulate the expression of C/EBPβ, STAT3, VEGFR1, FATP2 and S100A8. Lastly, the immunosuppressive activity of the induced MDSCs is inhibited by all-trans retinoic acid and STAT3 inhibitor BP-1-102 through cellular differentiation and dedifferentiation mechanisms, respectively. Together, our study establishes a human isogenic cell line system for neutrophils and MDSCs and this system is expected to facilitate future studies on the biology and therapeutics of human immunosuppressive neutrophils.

## 1. Introduction

In this new era of cancer immunotherapy, myeloid-derived suppressor cells (MDSCs) are increasingly recognized as a critically important immune cell population underlying the evasion of anti-tumor immunity and resistance to immunotherapy [1]. MDSCs are a heterogeneous population of immature myeloid cells that are expanded in various pathological conditions, especially in cancer [2]. Activation of MDSCs is usually manifested by increased expression of arginase 1 (ARG1) and inducible nitric oxide synthase (iNOS, aka NOS2), as well as increased production of reactive oxygen species (ROS) and reactive nitrogen species (RNS) [3]. It is now accepted that MDSCs employ a number of mechanisms to suppress both innate and adaptive immune responses in cancer [1,3]. MDSCs are classified as granulocytic or polymorphonuclear MDSCs (PMN-MDSCs) and monocytic MDSCs (M-MDSCs), based on morphological and phenotypical resemblance to neutrophils and monocytes, respectively. In mice, PMN-MDSCs and M-MDSCs are defined as CD11b^+^Ly6G^+^Ly6C^low^ cells and CD11b^+^Ly6G^−^Ly6C^high^ cells, respectively. In human, PMN-MDSCs and M-MDSCs are identified as CD11b^+^CD14^−^CD15^+^CD33^+^ cells and CD11b^+^CD14^+^CD15^−^CD33^+^ HLA-DR^−/low^ cells, respectively. According to the two-phase hypothesis of MDSC accumulation in cancer, granulocyte macrophage-colony stimulating factor (GM-CSF) and granulocyte-colony stimulating factor (G-CSF) belong to the first signals that drive the expansion of early MDSCs, whereas interleukin 6 (IL6) belongs to the second signals that mediate the acquisition of the immunosuppressive activity [2,4]. Increasing clinical evidence supports that MDSCs are abundant in human cancers including gliomas [5,6,7,8]. A meta-analysis shows that elevated MDSCs in the blood are an independent biomarker for overall survival, disease-free survival and progression-free survival in patients with various types of solid tumors [9].

Despite the functional importance and clinical relevance of MDSCs in cancer, clinically approved therapeutics targeting MDSCs remain an unachieved goal, partly because very few models of human MDSCs exist that are tractable to both mechanistic and therapeutic studies. Efforts have been made to generate in vitro cell line models for mouse and human MDSCs. Mouse CD11b^+^ Gr1^+^ myeloid cells were immortalized with oncogenes v-Myc and v-Raf to create cell lines with immunosuppressive activity [10], although the relevance of these oncogenes to MDSC biology is unclear. Mouse PMN-MDSCs and M-MDSCs could be induced from primary bone marrow cells incubated with GM-CSF alone or in combination with other cytokines, or with conditioned medium from mouse or human cancer cell lines [11,12,13,14,15]. Human CD33^+^HLA-DR^low^ MDSCs with an activated expression of iNOS, NDAPH oxidase and ARG1 could be induced by the co-culture of peripheral blood mononuclear cells (PBMCs) from healthy donors with various human cancer cell lines [16]. Taking a reductionist approach, the same group demonstrated that various combinations of tumor cell-secreted cytokines could induce normal PBMCs to become CD33^+^HLA-DR^low^ MDSCs with the GM-CSF and IL6 combination generating MDSCs with the highest consistency and potency [17]. We reason that an obvious limitation of the studies using freshly isolated bone marrow cells or PBMCs for human MDSC modeling is the difficulty in standardizing the procedure and achieving robust results independent of the donors. To address this problem, here we present the results where MDSCs are induced from a commonly used human promyelocytic cell line, HL60 [18], through dimethyl sulfoxide (DMSO) plus the cytokine cocktail GM-CSF and IL6. This new model will significantly promote the biological understanding and therapeutic discovery of MDSCs.

## 2. Results

### 2.1. GM-CSF and IL6 Interrupt the Induced Neutrophilic Differentiation of HL60 

**Figure 1:**
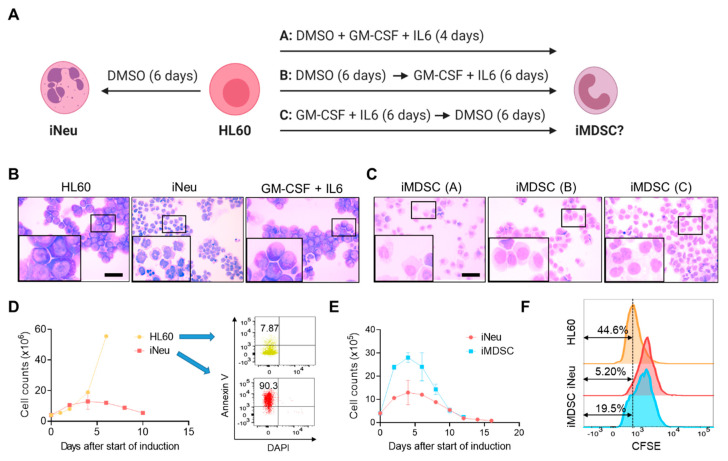
Induction of neutrophils and myeloid-derived suppressor cells (MDSCs) from HL60. (**A**) Experimental scheme for induced neutrophil (iNeu) and induced myeloid-derived suppressor cell (iMDSC) induction. Created with biorender.com. (**B**) Wright–Giemsa staining of HL60, iNeu, and HL60 treated with granulocyte macrophage-colony stimulating factor (GM-CSF) + interleukin 6 (IL6) for 6 days. Scale bar 50 μm. (**C**) Wright–Giemsa staining of iMDSC induced by the three conditions shown in (**A**). Scale bar 50 μm. (**D**) Proliferation curves and apoptosis analysis of HL60 and iNeu. Apoptotic cells were identified as Annexin V^+^ DAPI^−^ cells. (**E**) Proliferation curves of iNeu and iMDSC. (**F**) Representative carboxyfluorescein succinimidyl ester (CFSE) flow cytometer histograms showing the differentiation dilution of the CFSE labeling of HL60, iNeu and iMDSC on Day 4 after the start of induction.

It is a classical model to induce HL60 differentiation into neutrophils (iNeu) with polar-planer compounds such as DMSO [19]. How to induce HL60 to MDSCs (iMDSC) is unknown. Given that the GM-CSF and IL6 cytokine cocktail proved most effective to induce MDSC formation from PBMCs [17], we tested either simultaneous or sequential treatment of DMSO (1.25%) and GM-CSF+IL6 (10ng/mL each) in HL60, termed condition A, B and C respectively (Figure 1A). DMSO treatment alone for 6 days converted the nuclei from round shape in HL60 cells to a mixture of banded or segmented morphology in iNeu cells, confirmative of the neutrophilic maturation (Figure 1B). Moreover, GM-CSF+IL6 treatment alone did not change the morphology of HL60 (Figure 1B). On the contrary, when HL60 was treated with DMSO and GM-CSF+IL6 together or consecutively in either order, the cells showed decreased nucleocytoplasmic ratio and round or oval peripherally located nuclei (Figure 1C), suggesting that the cytokine cocktail interrupted the DMSO-induced differentiation of HL60 and the cells remained in an immature status. Considering that condition A only required 4 days whereas B and C needed 12 days, we chose to use the more rapid condition A subsequently for iMDSC induction from HL60. 

We compared the proliferation potential of HL60, iNeu and iMDSC. In contrast to the continued increase of cell number for HL60, iNeu cell numbers started to decline after Day 6 of DMSO induction and displayed massive apoptosis (Figure 1D). Compared with iNeu, iMDSC showed over a two-fold higher number of cells after induction; nevertheless, iMDSC also started to decline after Day 6 (Figure 1E). To track cell proliferation in a different way, we stained HL60, iNeu and iMDSC on Day 0 of induction with carboxyfluorescein succinimidyl ester (CFSE), and after 4 days, the fraction of CFSE^low^ cells showed the trend HL60 > iMDSC > iNeu (Figure 1F), consistent with the cell number measurement. Together, the results indicate that supplementing DMSO with GM-CSF and IL6 drives HL60 to a state distinct from differentiated neutrophils. 

### 2.2. iMDSC Express Surface Makers for Human MDSC Subsets

**Figure 2:**
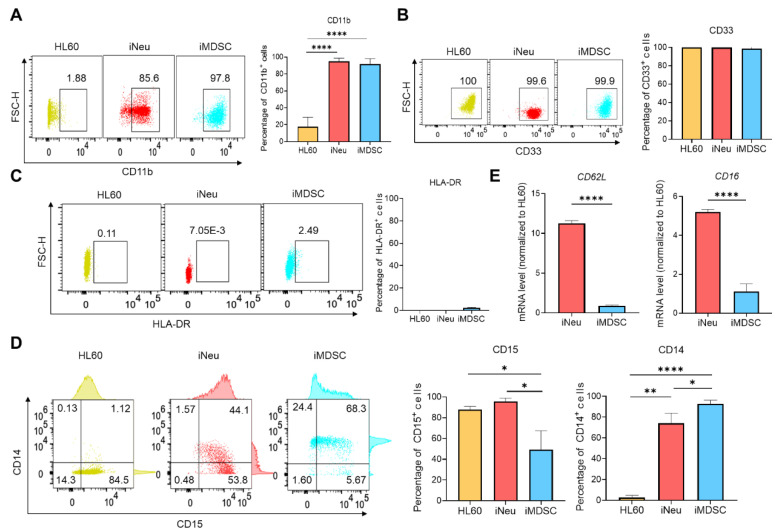
MDSC surface maker analysis for HL60, iNeu and iMDSC. (**A**–**D**) Flow cytometry analysis of CD11b, CD33, HLA-DR, CD14 and CD15 expression in HL60, iNeu and iMDSC. Both representative plots and average frequencies were shown. (**E**) Expression of *CD62L* and *CD16* at the mRNA level in HL60, iNeu and iMDSC, measured by qRT-PCR. Relative mRNA was normalized to *GAPDH* (internal control) and HL60 (the reference baseline of 1.0). * *p* < 0.05, ** *p* < 0.01, **** *p* < 0.0001, two-tailed Student’s *t*-test. For (**A**,**D**), one-way ANOVA followed by multiple comparisons returned equivalent significance levels.

Human MDSCs are characterized as CD11b^+^ CD33^+^ HLA-DR^−/low^ population and further divided into CD14^+^ M-MDSCs and CD15^+^ PMN-MDSCs [20]. We examined the surface marker expression pattern in HL60, iNeu and iMDSC. Both iNeu and iMDSC upregulated CD11b expression compared with HL60 (Figure 2A). All three cell states were positive for CD33 (Figure 2B) and negative for HLA-DR (Figure 2C). CD15/CD14 result was particularly interesting: HL60 were CD15^+^ CD14^−^, iNeu gained a slightly higher CD15 level with a small population becoming CD14^low^, and iMDSC displayed significant heterogeneity with the following subpopulations from high to low CD15^+^ CD14^+^ > CD15^−^ CD14^+^ > CD15^+^ CD14^−^ (Figure 2D). Consistent with the previous report on the lower level of CD16 and CD62L in PMN-MDSCs compared with steady-state neutrophils [21], *CD16* and *CD62L* transcripts were significantly lower in iMDSC than iNeu (Figure 2E). Together, we confirm that iNeu and iMDSCs are positive for the commonly used surface markers of human neutrophils and MDSCs, respectively. The heterogeneous CD15/CD14 expression pattern in iMDSC suggests that iMDSC comprise cells with features consistent with PMN-MDSCs and M-MDSCs. 

### 2.3. iMDSC Display Suppressive Activity toward Activated T Cells 

**Figure 3:**
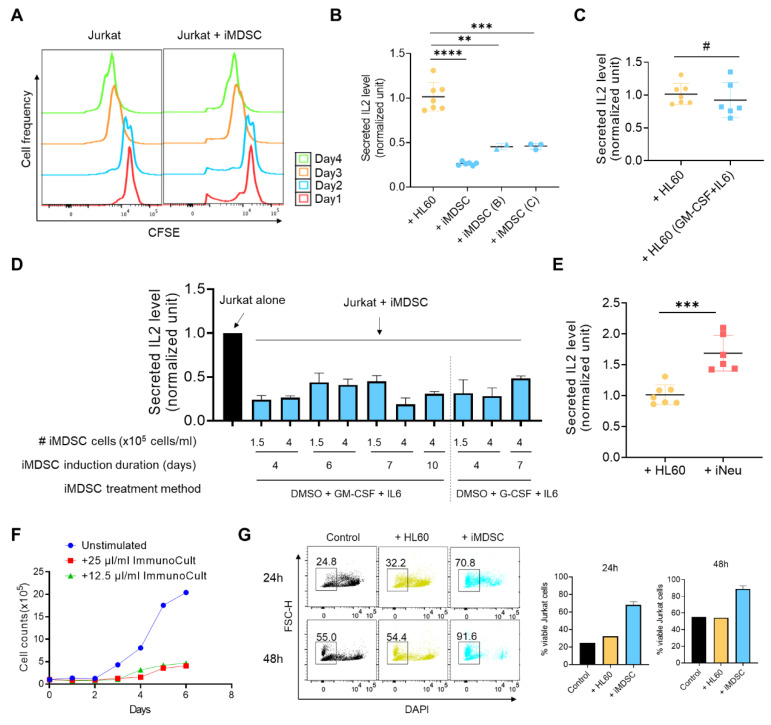
iMDSC suppress IL2 secretion and activation-induced cell death in stimulated Jurkat cells. (**A**) Representative CFSE flow cytometer histograms showing the proliferation-caused CFSE dilution of Jurkat cells alone or Jurkat cells co-cultured with iMDSCs during a 4-day incubation. (**B**) Relative IL2 level (measured by ELISA) in the medium from stimulated Jurkat cells co-cultured with HL60 or iMDSC (induced by conditions A, B and C as shown in Figure 1A). (**C**) Relative IL2 level (measured by ELISA) in the medium from stimulated Jurkat cells co-cultured with untreated HL60 or HL60 pre-treated with 10ng/mL GM-CSF + IL6 for 6 days. (**D**) Relative IL2 level (measured by ELISA) in the medium from stimulated Jurkat cells alone or co-cultured with iMDSC induced under indicated conditions. (**E**) Relative IL2 level (measured by ELISA) in the medium from stimulated Jurkat cells co-cultured with HL60 or iNeu. The results in (**B**–**E**) were normalized to the IL2 concentration secreted from ImmunoCult CD3/CD28-stimulated Jurkat alone. (**F**) Proliferation curves of unstimulated Jurkat or Jurkat stimulated by 25 μL/mL or 12.5 μL/mL ImmunoCult CD3/CD28 T Cell Activator. (**G**) Cell viability measurement with DAPI in flow cytometry of stimulated Jurkat alone, stimulated Jurkat co-cultured with HL60, or stimulated Jurkat co-cultured with iMDSC for 24h or 48h, respectively. Jurkat were stimulated by 25 μL/mL ImmunoCult CD3/CD28 T Cell Activator. In (**B**,**C**,**E**), ^#^ *p* > 0.05, ** *p* < 0.01, *** *p* < 0.001, **** *p* < 0.0001, two-tailed Student’s *t*-test. For (**B**), one-way ANOVA followed by multiple comparisons returned equivalent significance levels.

To define iMDSC as bona fide MDSCs, it is required to show that iMDSC suppress T cell activation. Inhibition of T cell proliferation (indicated by CFSE dilution) or inhibition of T cell cytokine production (IL2, IFNγ) are among the recommended functional assays to define MDSCs [20]. To unify the methodology of using human cell lines instead of primary cells like PBMCs in our study, our T cell model of choice was the CD3/CD28 antibody-stimulated Jurkat cells, a widely used immortalized T lymphocyte cell line [22]. Co-culture of iMDSC with CFSE-labeled Jurkat cells did not affect CFSE dilution (Figure 3A), presumably due to the stimulus-independent high proliferative ability of Jurkat which made the CFSE dilution assay unsuitable to measure iMDSC activity. However, when we co-cultured iMDSC induced by the 3 conditions (Figure 1A) with CD3/CD28 antibody-stimulated Jurkat cells at the ratio of 1:1, we found that all three MDSC induction conditions inhibited IL2 secretion compared with co-culture with HL60, quantified with ELISA (Figure 3B). Among the 3 conditions, the most rapid condition A chosen as default for iMDSC induction elicited strongest suppression on IL2 production from Jurkat. When CD3/CD28 antibody-stimulated Jurkat cells were cultured with iMDSC conditional medium, IL2 production was unaffected (data not shown), suggesting that iMDSC suppression is mediated by cell–cell contact. No suppressive activity was observed from HL60 pre-treated with GM-CSF+IL6 for 6 days (Figure 3C), reinforcing that differentiation inducer DMSO is required for iMDSC induction from HL60. A time point analysis showed that iMDSC taken through Day 4 to Day 10 of the induction course consistently sustained the activity to inhibit IL2 production from stimulated Jurkat cells (Figure 3D). Interestingly, G-CSF could replace GM-CSF in the iMDSC induction cocktail to achieve a comparable level of immunosuppression (Figure 3D). In contrast to the immunosuppressive activity of iMDSC, iNeu enhanced IL2 production from stimulated Jurkat cells (Figure 3E), highlighting the distinct function of neutrophils and MDSCs [23]. 

Activation-induced cell death (AICD) is a phenomenon in T cells (e.g., Jurkat) stimulated in vitro with anti-CD3 or phorbol esters [24], and its main mechanism is protein kinase C theta activation and Fas ligand expression [25]. Consistently, we observed decreased numbers of Jurkat cells after anti-CD3/anti-CD28 stimulation (Figure 3F). We hypothesized that iMDSC, but not HL60, would attenuate AICD due to the activity of iMDSC to suppress Jurkat activation. Indeed, we observed a much higher frequency of viable stimulated Jurkat cells when Jurkat cells were co-cultured with iMDSC in comparison to culture alone or co-culture with HL60 (Figure 3G). Taken together, iMDSC, but not HL60 or iNeu, exhibit potent activity to suppress IL2 production and AICD in Jurkat cells, validating their identity as MDSC. 

### 2.4. iMDSC Selectively Upregulate the Expression of MDSC Functional Players 

**Figure 4:**
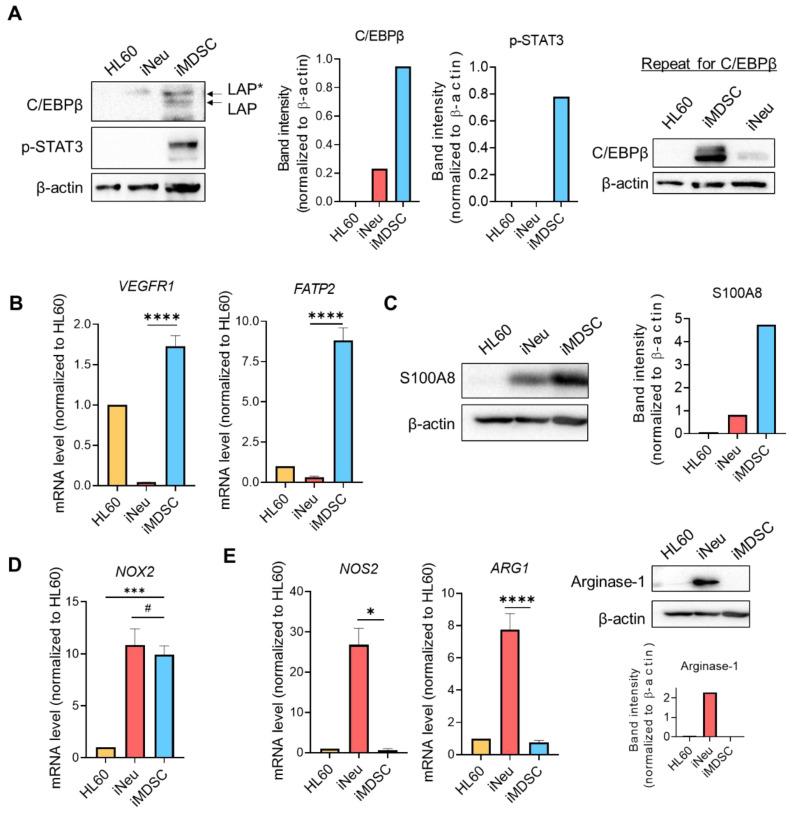
iMDSC selectively upregulate the expression of MDSC functional players. (**A**) Protein levels of C/EBPβ and phospho-STAT3 (Tyr705) in HL60, iNeu and iMDSC, detected by Western blot. The normalized band intensity was shown. Shown on the right is the repeat experiment for C/EBPβ with independently extracted cell lysates. (**B**) Expression of *VEGFR1* and *FATP2* (measured by qRT-PCR) in HL60, iNeu and iMDSC. Relative mRNA was normalized to *GAPDH* (internal control) and HL60 (the reference baseline of 1.0). (**C**) Protein level of S100A8 in HL60, iNeu and iMDSC, detected by Western blot. The normalized band intensity was shown. (**D**) Expression of *NOX2* (measured by qRT-PCR) in HL60, iNeu and iMDSC. Relative mRNA was normalized to *GAPDH* (internal control) and HL60 (the reference baseline of 1.0). (**E**) Expression of *NOS2* and *ARG1* at the mRNA level measured by qRT-PCR, and Arginase-1 at the protein level detected by Western blot in HL60, iNeu and iMDSC. In (**B**,**D**,**E**), ^#^ *p* > 0.05, * *p* < 0.05, *** *p* < 0.001, **** *p* < 0.0001, two-tailed Student’s *t*-test. For (**D**), one-way ANOVA followed by multiple comparisons returned equivalent significance levels.

Having confirmed the immunosuppressive activity of iMDSC, we wanted to compare the expression pattern in HL60, iNeu and iMDSC of some genes known to mediate MDSC function. MDSC expansion and activation are orchestrated by cytokine-activated transcription factors, including CCAAT enhancer-binding protein β (C/EBPβ) and signal transducer and activator of transcription 3 (STAT3) [26]. Stimulated by cytokines including GM-CSF and IL6, C/EBPβ plays an indispensable role in emergency granulopoiesis and expansion of MDSCs in cancer [15,27]. C/EBPβ has three isoforms: liver-enriched activator proteins LAP*, LAP, and liver-enriched inhibitory protein LIP [28]. Among these isoforms, LAP* and LAP function as transcriptional activators of inflammation-linked genes, such as IL-6, TNF, and G-CSF, whereas LIP antagonizes C/EBPβ transcriptional activity through a dominant-negative mechanism. In our cell system, C/EBPβ LAP* was slightly elevated in iNeu compared with HL60, and both LAP* and LAP were dramatically upregulated in iMDSC (Figure 4A). STAT3 is also critical for the expansion and activation of MDSCs, especially PMN-MDSCs [29,30]. Consistent with the result of C/EBPβ, STAT3 phosphorylation was virtually absent in HL60 and iNeu, but massively increased in iMDSC (Figure 4A). 

Next, we examined the expression of a few previously identified genes that facilitate MDSC expansion or execute the immunosuppressive function of MDSCs. Vascular endothelial growth factor receptor 1 (VEGFR1) is upregulated in both human and mouse PMN-MDSCs and mediate VEGF-dependent MDSC expansion and infiltration to the tumor bed [21,31]. Fatty acid transport protein 2 (FATP2) is recently identified to be exclusively upregulated by both human and mouse PMN-MDSCs compared with neutrophils [32]. FATP2 is activated by the GM-CSF/STAT5 signaling axis and promotes arachidonic acid uptake to enhance MDSC immunosuppression [32]. In our cell system, both *VEGFR1* and *FATP2* were significantly upregulated in iMDSC relative to iNeu at the mRNA level (Figure 4B). S100 Calcium Binding Protein A8 and A9 (S100A8/A9) belong to damage-associated molecular pattern molecules and are known to be upregulated in PMN-MDSCs and M-MDSCs [33,34]. S100A8/A9 sustained MDSC level in vivo*,* and peptibody targeting S100A8/A9 depleted both MDSC subsets in mice [33,35]. Consistent with these reports, we detected a higher level of S100A8 in iMDSC than iNeu (Figure 4C). 

MDSC inhibit T cell effector functions through a range of mechanisms, with some widely recognized ones such as ARG1-mediated depletion of L-arginine, and NOS2 and NADPH oxidase 2 (NOX2) production of ROS and RNS [1]. Among these 3 genes, *NOX2* expression level was significantly elevated in both iNeu and iMDSC compared with HL60, but there was no difference between iNeu and iMDSC (Figure 4D). By contrast, *NOS2* and *ARG1* were exclusively upregulated in iNeu (Figure 4E). Together, these results suggest that iMDSC may employ mechanisms such as FATP2 rather than the conventionally recognized mechanisms (like L-arginine depletion or ROS/RNS production) to suppress the T cell functions. 

### 2.5. All-Trans Retinoic Acid (ATRA) and STAT3 Inhibitor BP-1-102 Direct iMDSC to Distinct Non-Suppressive States

**Figure 5:**
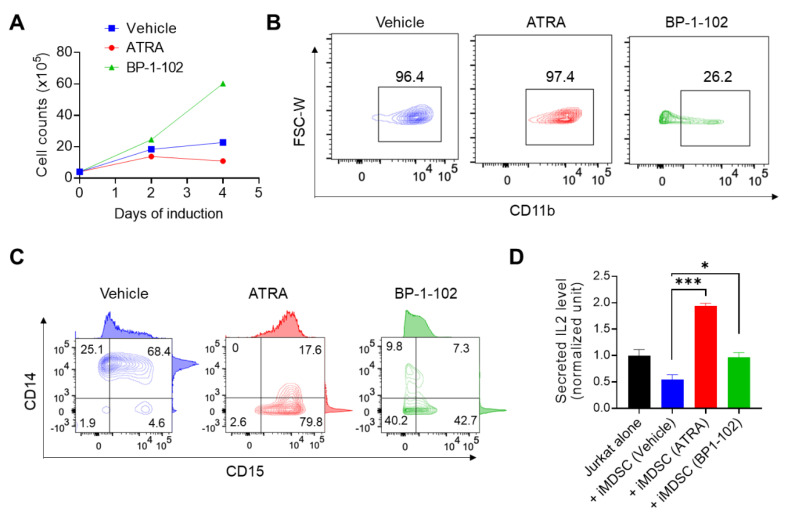
All-trans retinoic acid (ATRA) and STAT3 inhibitor BP-1-102 direct iMDSC to distinct non-suppressive states. (**A**) Proliferation curves of iMDSC cells treated with vehicle, ATRA and BP-1-102 throughout the 4-day induction course. (**B**,**C**) Flow cytometry analysis of CD11b, CD14 and CD15 expression on iMDSC treated with vehicle, ATRA and BP-1-102 at the end of the 4-day induction course. (**D**) Relative IL2 level (measured by ELISA) in the medium from stimulated Jurkat alone or co-cultured with iMDSC pre-treated with vehicle, ATRA and BP-1-102. * *p* < 0.05, *** *p* < 0.001, two-tailed Student’s *t*-test. For (**D**), one-way ANOVA followed by multiple comparisons returned equivalent significance levels.

We wanted to provide the proof of concept evidence on the validity of iMDSC as a platform for the discovery of pharmacological inhibitors of MDSCs. We focused on two targets closely implicated in MDSC therapeutics. ATRA is an active metabolite of vitamin A and is used as a differentiation therapy for the treatment of acute promyelocytic leukemia [36]. Concordantly, ATRA is active to induce the granulocytic differentiation of HL60, a cell line derived from acute promyelocytic leukemia [37]. ATRA directs MDSC differentiation to mature dendritic cells, macrophages, and granulocytes [38,39], which has prompted several clinical trials with promising results showing that the effect of ATRA on MDSCs could enhance immunotherapy in cancer patients (e.g., NCT02403778) [40]. Mechanistically, ATRA is known to decrease the frequency and function of MDSCs through activation of extracellular signal–regulated kinase 1/2, upregulation of glutathione synthase and production of glutathione [41]. As stated above, STAT3 is critical for the activation of immunosuppressive myeloid cells including MDSCs [42]. STAT3 inhibitors or STAT3-targeted oligonucleotide agents reduce the number and abolish the immunosuppressive activity of MDSCs in mouse models and cancer patients [30,43,44,45]. We tested the effect of ATRA and a STAT3 inhibitor BP-1-102 [46] on iMDSC differentiation from HL60 by including these two agents in the condition A (DMSO + GM-CSF + IL6) of HL60 differentiation. We observed the opposite effect of these two agents: ATRA decreased whereas BP-1-102 increased the number of cells in culture (Figure 5A). On Day 4 of culture, we analyzed the surface marker expression of the cells. For CD11b, both vehicle and ATRA treated cells were CD11b^+^, but BP-1-102 decreased CD11b^+^ cells to only about 25% (Figure 5B). For CD15/CD14, ATRA shifted the heterogeneous iMDSC population to a population dominated by CD15^+^ CD14^−^ cells (similar to iNeu), whereas BP-1-102 enriched CD15^−^CD14^−^ undifferentiated cells to about 40% (Figure 5C). Importantly, when the differently treated cells were washed off the drug and added to simulated Jurkat cells for the immunosuppression assay, both ATRA and BP-1-102 abrogated the ability of the treated iMDSC to suppress IL2 production (Figure 5D). In fact, ATRA treated cells functionally resembled iNeu (Figure 3E) and enhanced Jurkat IL2 production (Figure 5D). Taken together, ATRA and BP-1-102 exerted differential effects on iMDSC: ATRA overrode the effect of GM-CSF and IL6 in the condition A and together with DMSO drove HL60 to non-suppressive neutrophils (i.e., iNeu-like), while BP-1-102 overruled the effect from all factors of the iMDSC induction condition (DMSO + GM-CSF + IL6) and returned the cells to the progenitor status (i.e., HL60-like). Through distinct mechanisms, both agents abrogate the immunosuppressive activity of iMDSC and support that iMDSC is a valid model for finding other MDSC-targeted therapeutics. 

## 3. Discussion

Our study established a rapid (4-day) in vitro model to generate isogenic human neutrophils and MDSCs from the widely used myeloid progenitor cell line HL60. HL60 is a popular cell line to study neutrophilic differentiation. MDSC-like cells were successfully generated from HL60 by the combination treatment with DMSO, GM-CSF and IL6. Similar to the heterogeneous nature of spontaneously formed MDSCs in patients, iMDSCs were uniformly CD11b^+^ CD33^+^ HLA-DR^−/low^, yet heterogeneous for CD15 and CD14 expression (Figure 2), indicating they were a mixture of PMN-MDSCs and M-MDSCs. It is not our intention to claim that this model outperforms the previously published human bone marrow or PBMC-based MDSC models [15,16,17]. However, we do believe that this HL60-based neutrophil and MDSC model will lower the technical and logistical barriers and allow more researchers from non-medical institutions (like ours) to study MDSC differentiation and therapeutics, as well as the distinction between neutrophils and MDSCs. A limitation of the current study is that we only focused on the cytokine combination of GM-CSF and IL6 based on the notion that these two cytokines are known inducers of MDSCs from PBMCs with strong activity and consistency [17]. Further studies are warranted to investigate whether and how MDSCs can be induced from HL60 cells by other tumor-derived cytokines, metabolites or even extracellular vesicles like exosomes. 

An intriguing property of iMDSC is their lack of *NOS2* and *ARG1* expression (Figure 4E), suggesting that L-arginine depletion or RNS production are unlikely mechanisms to explain their activity to suppress IL2 production from T cells. Because both iNeu and iMDSC upregulated *NOX2* expression (Figure 4D) but these two populations displayed opposite effect on T cell IL2 production (Figure 3), we argue that ROS is also unlikely the key to iMDSC immunosuppression. Therefore, iMDSC provides the opportunity to unravel potentially unconventional mechanisms of immunosuppression by MDSCs in future studies. Notwithstanding, it is important to recognize the caveat that any of these unconventional mechanisms likely involve unconventional intracellular regulatory molecules, which might be identified as candidate therapeutic targets in a functional screening study but may not be operative in MDSCs isolated directly from cancer patients or induced from primary human bone marrow or PBMCs. Therefore, the iMDSC system at best only models some of the relevant immunosuppressive functions that MDSCs exert in vivo, and validation studies following functional screens will be critical. We speculate that high *FATP2* expression in iMDSC (Figure 4B) is likely a major contributor to the acquisition of immunosuppressive activity through the uptake of arachidonic acid and the synthesis of prostaglandin E_2_, based on the recent study from the Gabrilovich group [32]. 

ATRA and the STAT3 inhibitor pushed iMDSCs toward the two edges of the differentiation spectrum: ATRA induced the cells to become fully differentiated neutrophils whereas BP-1-102 dedifferentiated the cells to become like myeloid progenitors. In both cases, the cells lost the immunosuppressive activity, suggesting that a key strategy of MDSC-targeted therapy in patients should pharmacologically unlock the status of MDSCs and mobilize them toward either end of the myeloid differentiation spectrum. We envision that the approaches following this principle could be more successful clinically than methods aimed at killing MDSCs due to the preserved myeloid cell pool thus the expected lower toxicity. One limitation of the HL60-based iMDSC system is the interference of MDSC biology and therapy by the genetic alterations in HL60, such as *NRAS* and *CDKN2A* point mutations, *MYC* amplification and *TP53* deletion [47,48,49]. Therefore, the HL60 system should be treated as an early phase discovery tool (e.g., screen for MDSC inhibitors) and the conclusions should be validated with MDSCs differentiated from normal bone marrow cells or PBMCs or MDSCs isolated from cancer patients. 

## 4. Materials and Methods 

### 4.1. Cell Culture and Induced Differentiation

HL60 cells (ATCC, CCL-240, Manassas, VA, USA) were cultured in Iscove’s Modified Dulbecco’s Medium (IMDM, HyClone, GE Healthcare Life Sciences, SH30228.01, Marlborough, MA, USA) supplemented with 20% fetal bovine serum (FBS, HyClone, GE Healthcare Life Sciences SH30396.03, Marlborough, MA, USA) and 1X Penicillin/Streptomycin (Caisson Labs, PSL01, Smithfield, UT, USA). Jurkat cells (clone E6-1, ATCC, TIB-152, Manassas , VA, USA) were cultured in IMDM supplemented with 10% FBS and 1X Penicillin/Streptomycin. Cells were verified to be mycoplasma free by PCR-based test every two weeks. Neutrophils were induced from HL60 by adding 1.25% DMSO (Sigma-Aldrich, D4540, St. Louis, MO, USA) for 6 days. MDSCs were induced from HL60 by culturing the cells in the presence of 1.25% DMSO, 10ng/mL GM-CSF (Tonbo Biosciences, 21-8339-U020, San Diego, CA, USA), and 10ng/mL IL6 (Tonbo Biosciences, 21-8069-U020, San Diego, CA, USA). HL60, iNeu, and iMDSC were cultured between 1 × 10^5^ cells/mL and 1 × 10^6^ cells/mL.

### 4.2. Flow Cytometry, Wright–Giemsa Staining, and Western Blot

Cells (1 × 10^6^) were incubated with diluted Fc block (1:40, Miltenyi Biotec, 130-059-901, Auburn, CA, USA) for 30 min on ice. Cells were then stained with antibodies including anti-CD11b-APC (Biolegend, 101211, San Diego, CA, USA), anti-CD11b-PerCP-Cy5.5 (Tonbo Biosciences, 65-0112-U100, San Diego, CA, USA), anti-HLA-DR-PE (Tonbo Biosciences, 50-9952-T025, San Diego, CA, USA), anti-CD15-Alexa Fluor 488 (Biolegend, 301910, San Diego, CA, USA), and anti-CD14-PE-Cy7 (Tonbo Biosciences, 60-0149-ST05, San Diego, CA, USA). CD33^+^ cells were stained with anti-CD33 (Biolegend, 303402, San Diego, CA, USA) and the secondary antibody anti-mouse IgG-Alexa Fluor 647 (Jackson ImmunoResearch Laboratories, 115-605-146, West Grove, PA, USA). Apoptotic cells and dead cells were distinguished by Annexin V (Tonbo Biosciences, 20-6409-T100, San Diego, CA, USA) and DAPI (0.5 μg/mL) double staining. Flow cytometry analyses were performed on BD LSRFortessa X-20 flow cytometer. Quantification and data presentation were done with FlowJo Software version 10.4. For Wright–Giemsa staining, cells were spun down on a glass slide by Cytospin centrifuge (Thermo Fisher Scientific, Waltham, MA, USA) and air-dried. The slide was incubated with 1 mL Wright–Giemsa staining buffer (VWR, 101411-114, Radnor, PA, USA) for 4 min and then incubated with 2 mL distilled water for 8 min followed by rinsing with distilled water. For Western blot, the standard procedure as we recently described was followed [50]. The primary antibodies used included C/EBPβ (Abcam, ab32358), Phospho-Stat3 (Tyr705) (CST, 9145, Danvers, MA, USA), β-actin (Santa Cruz Biotechnology, sc-47778, Dallas, TX, USA), S100A8 (Proteintech, 15792-1-AP, Rosemont, IL, USA), and Arginase-1 (CST, 93668S, Danvers, MA, USA).

### 4.3. HL60, iNeu and iMDSC Proliferation by Cell Counting and CFSE Labeling

HL60 (4 × 10^5^ cells/mL, 2 mL) were seeded to each well of 6-well plates. Inducers were added on Day 0 to generate iNeu and iMDSCs. The induction medium was refreshed every two days. The cell numbers were counted every two days by hemocytometer. The cell viability was confirmed by Trypan blue solution (VWR, 45000-717, Radnor, PA, USA). For carboxyfluorescein diacetate succinimidyl ester (CFSE; Invitrogen, C1157, Thermo Fisher Scientific, Waltham, MA, USA) labeling experiment, HL60 were stained with 5 μM solution of CFSE for 5 min at room temperature, then washed with PBS (Caisson Labs, PBL01, Smithfield, UT, USA) containing 5% FBS for 3 times, then plated at 20,000 cells in 200 μL per well in complete medium in 96-well plate. Inducers were added on Day 0 to generate iNeu and iMDSCs. Cells were continued in culture until Day 4 to be analyzed for the viability and CFSE fluorescence intensity on BD LSRFortessa X-20 flow cytometer.

### 4.4. Jurkat Cell Proliferation Assessed by CFSE Labeling

Jurkat cells (1 × 10^6^ cells/mL) were stained with 5 μM CFSE for 5 min at room temperature and washed with PBS containing 5% FBS for 3 times, then stimulated with ImmunoCult Human CD3/CD28 T Cell Activator (STEMCELL Technologies, 10971, Cambridge, MA, USA). HL60, iNeu or iMDSC were added to Jurkat at 1:1 ratio and incubated for 4 days in IMDM containing 10% FBS in 96-well flat-bottomed culture plates. At the endpoint, CFSE fluorescence intensity was quantified for CD3^+^ cells by flow cytometry on BD LSRFortessa X-20 flow cytometer.

### 4.5. IL2 Production Assay to Measure T Cell Suppression 

The stimulation of Jurkat and the co-culture of Jurkat with HL60, iNeu or iMDSC were set up as stated above. Cells were harvested 24 h later and centrifuged at 450 g at 4 °C for 10 min. The culture supernatant was stored at −80 °C until use. The supernatant was used to quantify secreted IL2 concentration with Human IL-2 ELISA kit (Biolegend, 431804, San Diego, CA, USA) per the manufacturer’s instructions.

### 4.6. Quantitative Reverse Transcription-Polymerase Chain Reaction (qRT-PCR)

Total RNA was isolated using a Total RNA Isolation Miniprep Kit (Bio Basic, BS1361, BUFFALO, NY, USA). Complementary DNA was synthesized using an All-In-One RT MasterMix (ABM, G490, Richmond, BC, Canada) according to the manufacturer’s instruction. Relative quantities of specific mRNA species were measured using SYBR Green qPCR Master Mix (Bimake, B21203, Houston, TX, USA) on CFX Connect Real-Time PCR System (Bio-Rad, Hercules, CA, USA). Amplification was performed with 40 cycles at 95 °C for 15 s and 60 °C for 30 s. Data was analyzed with the 2^-ΔΔ*CT*^ method with the genes of interest normalized to GAPDH (housekeeping control). The primer sequences were as follows: *NOX2* (forward: ACC GGG TTT ATG ATA TTC CAC CT; reverse: GAT TTC GAC AGA CTG GCA AGA); *NOS2* (forward: TTC AGT ATC ACA ACC TCA GCA AG; reverse: TGG ACC TGC AAG TTA AAA TCC C); *ARG1* (forward: GTG GAA ACT TGC ATG GAC AAC; reverse: AAT CCT GGC ACA TCG GGA ATC); *VEGFR1* (forward: TTT GCC TGA AAT GGT GAG TAA GG; reverse: TGG TTT GCT TGA GCT GTG TTC); *FATP2* (forward: CAG TGA TCT TGG TGT CCT GTA T; reverse: CTT TCA GCA CAT TGC TGA TTA CCT); *CD62L* (forward: ACC CAG AGG GAC TTA TGG AAC; reverse: GCA GAA TCT TCT AGC CCT TTG C); *CD16* (forward: CCT CCT GTC TAG TCG GTT TGG; reverse: TCG AGC ACC CTG TAC CAT TGA).

### 4.7. Treatment with ATRA and BP1-102

HL60 (4 × 10^5^ cells/mL, 2 mL) was seeded to each well of 6-well plates. Inducers were added on Day 0 to generate iMDSCs. Meanwhile, on Day 0, 1 μM ATRA (Cayman Chemical Company, 11017, Ann Arbor, Michigan, USA) or 6.8 μM BP-1-102 (Calbiochem, 573132, MilliporeSigma, MA, USA) was added into the iMDSC induction medium. The medium was refreshed every two days. The cell numbers were counted every two days. After 4-day incubation, ATRA-treated iMDSC and BP-1-102-treated iMDSC were washed off of the inhibitors and used for the flow cytometry analysis to examine their surface markers or co-cultured with Jurkat cells to examine their immunosuppressive activity.

### 4.8. Statistical Analysis

Data were displayed as mean ± standard deviation. Experiments were repeated at least three times with representative results shown. Comparisons between two groups were performed using the unpaired Student’s t-test with GraphPad Prism software v8.2 (GraphPad Software, San Diego, CA, USA). *p* < 0.05 was considered statistically significant.

## 5. Conclusions

HL60 cell line is induced by DMSO to differentiate into mature neutorphils or induced by the combination treatment of DMSO, GM-CSF and IL6 to become CD11b^+^ CD33^+^ HLA-DR^−/low^ MDSC-like cells. The induced MDSCs suppress IL2 production from Jurkat cells and upregulate the expression of C/EBPβ, STAT3, VEGFR1, FATP2 and S100A8. The immunosuppressive activity of the induced MDSCs are inhibited by all-trans retinoic acid or STAT3 inhibitor.

## Abbreviations

ARG1	arginase 1
ATRA	all-trans retinoic acid
C/EBPβ	CCAAT enhancer binding protein β
CD11b	cluster of differentiation molecule 11B
CDKN2A	cyclin-dependent kinase inhibitor 2A
CFSE	carboxyfluorescein succinimidyl ester
DMSO	dimethyl sulfoxide
FATP2	fatty acid transport protein 2
FBS	fetal bovine serum
G-CSF	granulocyte-colony stimulating factor
GM-CSF	granulocyte macrophage-colony stimulating factor
HLA-DR	Human Leukocyte Antigen – DR isotype
IL2	interleukin 2
IL6	interleukin 6
IMDM	Iscove’s Modified Dulbecco’s Medium
iMDSC	induced myeloid-derived suppressor cell
iNeu	induced neutrophil
iNOS	inducible nitric oxide synthase
MDSC	myeloid-derived suppressor cell
M-MDSC	monocytic MDSC
NOX2	NADPH oxidase 2
NRAS	Neuroblastoma RAS viral oncogene homolog
PBMCs	peripheral blood mononuclear cells
PMN	polymorphonuclear
PMN-MDSC	polymorphonuclear MDSC
RNS	reactive nitrogen species
ROS	reactive oxygen species
S100A8	S100 calcium binding protein A8
STAT3	signal transducer and activator of transcription 3
VEGFR1	vascular endothelial growth factor receptor 1
